# Design and feasibility of smartphone-based digital phenotyping for long-term mental health monitoring in adolescents

**DOI:** 10.1371/journal.pdig.0000883

**Published:** 2025-07-01

**Authors:** Debbie Huang, Patrick Emedom-Nnamdi, Jukka-Pekka Onnela, Anna Van Meter

**Affiliations:** 1 Department of Biostatistics, Harvard T.H. Chan School of Public Health, Boston, Massachusetts, United States of America; 2 Department of Health Science, California State University Long Beach, Long Beach, California, United States of America; 3 Department of Child and Adolescent Psychiatry, NYU Grossman School of Medicine, New York, New York, United States of America; Jordan University of Science and Technology, JORDAN

## Abstract

Assessment of psychiatric symptoms relies on subjective self-report, which can be unreliable. Digital phenotyping collects data from smartphones to provide near-continuous behavioral monitoring. It can be used to provide objective information about an individual’s mental state to improve clinical decision-making for both diagnosis and prognostication. The goal of this study was to evaluate the feasibility and acceptability of smartphone-based digital phenotyping for long-term mental health monitoring in adolescents with bipolar disorder and typically developing peers. Participants (aged 14–19) with bipolar disorder (BD) or with no mental health diagnoses were recruited for an 18-month observational study. Participants installed the Beiwe digital phenotyping app on their phones to collect passive data from their smartphone sensors and thrice-weekly surveys. Participants and caregivers were interviewed monthly to assess changes in the participant’s mental health. Analyses focused on 48 participants who had completed participation. Average age at baseline was 15.85 years old (SD = 1.37). Approximately half (54%) identified as female, and 54% identified with a minoritized racial/ethnic background. Completion rates across data types were high, with 99% (826/835) of clinical interviews completed, 89% of passive data collected (22,233/25,029), and 47% (4,945/10,448) of thrice-weekly surveys submitted. The proportion of days passive data were collected was consistent over time for both groups; the clinical interview and active survey completion decreased over the study course. Results of this study suggest digital phenotyping has significant potential as a method of long-term mental health monitoring in adolescents. In contrast to traditional methods, including interview and self-report, it is lower burden and provides more complete data over time. A necessary next step is to determine how well the digital data capture changes in mental health to determine the clinical utility of this approach.

## Author summary

This is the first study to collect and report digital phenotyping data from adolescent patients and community controls followed for longer than a year. Digital phenotyping provides more complete data than traditional assessment methods over time. Results of this study suggest digital phenotyping could be a practical, nonburdensome way to monitor mental health in adolescents.

Bipolar disorder (BD) is associated with significant mortality and morbidity. It typically begins in adolescence or early adulthood, an important developmental period during which higher education, first jobs, and relationships are pursued [1]. Recurrent mood episodes during this period can have a devastating impact on a young person’s ability to achieve a high quality of life as an adult. Close clinical monitoring can help enable swift intervention when a patient’s mental health deteriorates [2–4]. However, adolescents are among the populations most difficult to engage in ongoing mental health care [5–7]. Additionally, traditional monitoring, despite being expensive and time consuming for families, is often insufficient - typical psychiatry appointments, which occur on a monthly or quarterly basis, are usually not frequent enough to capture changes before a young person experiences some consequences of relapse (e.g., academic challenges, peer difficulties, hospitalization). A low burden method by which to predict the onset of a new mood episode would create an opportunity to intervene and reduce exposure to the harmful effects of recurrent episodes.

Digital phenotyping - the “moment-by-moment quantification of the human phenotype in situ” using data collected from smartphone sensors (accelerometer, texts, calls, GPS) – may make this possible [8–10]. A recent systematic review of 29 studies that employed digital phenotyping to monitor symptoms concluded that using data collected from smartphones and wearable devices is a feasible approach for assessing mental health status across people with mental health disorders [11]. While digital phenotyping has been used to identify mood changes and potential signs of relapse in adults with BD, it has not yet been applied to adolescents [12–14]. Adolescents may be an ideal population in which to utilize this approach; they are highly engaged with their smartphones and find digital mental health tools more appealing than in-person services [15,16].

Digital phenotyping has the potential to offer new insights about mental health disorders, particularly through longitudinal monitoring. However, the realization of its potential is dependent on whether adolescent participants are willing and able to engage with the approach (i.e., feasibility and acceptability) and whether the collected data provide useful information (i.e., data quality).

The primary goal of this study is to evaluate the feasibility and acceptability of digital phenotyping as a method of long-term monitoring in adolescents with BD and typically-developing (TD) peers. A secondary goal is to compare the completeness and quality of data collected using digital phenotyping to data collected using traditional methods of assessment including participant self-reported mood (requested three times a week on the smartphone). We hypothesized that participants would adhere to the digital phenotyping protocol for the duration of the 18-month study period and that the completeness and quality of the digital phenotyping data would be superior to the data collected through traditional means of interviews and self-reports, particularly in the later months of the follow-up period. Furthermore, we hypothesized that there would be no significant differences in the completeness or quality of data between the BD and typically developing groups.

## Method

### Ethics statement

All study procedures were approved by the Institutional Review Boards of Northwell Health and NYU Langone Health. Informed written consent was obtained from all caregivers and written assent/consent was obtained from all participants (depending on age).

### Participants

This study included the first 48 enrolled participants, all of whom had completed the full 18-months of participation. Adolescents (aged 14–19) with bipolar disorder (subtype I, II, or other specified BD as defined by the Course & Outcome of Bipolar Youth study BP NOS criteria [17]) were recruited through inpatient and outpatient psychiatric services at a large northeastern healthcare organization. Only 1% of adolescents approached for the study were ineligible because they did not own a smartphone.

### Measures

Participants and caregivers completed a series of online questionnaires using REDCap at baseline and at each follow-up assessing symptoms of mania, depression, and anxiety, among other symptoms and behaviors of interest. This study used the open-source high-throughput Beiwe platform for smartphone-based collection of active (surveys) and passive (phone logs and sensor) data [18]. Passive features were collected continuously and included smartphone usage logs (e.g., turning on/unlocking phone screen and battery level), GPS, and accelerometer data in their raw unprocessed form, among others. The thrice-weekly surveys asked about mood, sleep, and recent activities. In addition to providing valuable information, the surveys help to keep the app active, ensuring continuous passive data collection. This is necessary because the Apple iOs is designed to prioritize frequently used applications to preserve battery life and space, applications with which the user is not frequently engaged may be deactivated.

The active data *study* completion rate was calculated as the sum of the total number of surveys completed by all participants divided by the sum of the total number of surveys delivered to all participants by the Beiwe app while the participants were enrolled. Quality of the active data was assessed by survey duration, which is the time between when the survey was initiated or opened by the participant and when it was submitted. Very fast (i.e., < 15 seconds, which is less time than it took to read the questions) or very long (i.e., > 5 minutes, which is five times longer than average and could mean individual question responses within the same survey reflected different states of mind) responses were considered poor quality.

The passive data *study* completion rate was calculated as the total number of days when some passive phone data (>0 bytes) were collected by all participants divided by the total number of actual participation days (excluding any days post-dropout) of all participants. A natural way to assess the completeness of passive data collection is through the volume of collected data [19]. In this study, we examined the average daily data volume, which was calculated for a given data type (e.g., GPS) by dividing the total completion proportion by the days each participant was in the study, and subsequently averaging these individual results across all participants.

Clinical interview completion rate was calculated as the sum of the total number of clinical interviews completed by all participants divided by the total number of clinical interviews scheduled.

### Procedures

Study information was provided to clinicians, so they could refer patients with bipolar disorder. Additionally, medical records were reviewed to identify patients who met study criteria and research staff contacted patient caregivers to inform them about the study. Typically developing participants (no current psychiatric disorder, no lifetime mood/psychotic disorder, and no family history of BD) were recruited through pediatricians’ offices and extra-curricular activities.

Depending on the family’s preference and current COVID-19 pandemic-related restrictions, the baseline assessment occurred in person or over video conference. Although COVID-19 affected all aspects of life, our decision to accommodate family preferences by offering virtual appointments throughout the study – not just during the lockdown period – reduces the likelihood of systematic biases affecting who participated. The baseline appointment included a structured clinical interview with the participant and caregiver to assess lifetime history of mental health disorders and to verify inclusion and exclusion criteria, a neuropsychological exam, a REDCap-based survey of study questionnaires, and installation of the Beiwe study app on their personal smartphone.

Participants and caregivers were interviewed monthly using the mood modules from the KSADS [20] and the Psychiatric Status Rating Scale [21]. They also completed monthly follow-up surveys about the adolescent’s mood (youth- and caregiver report forms of the 10-item General behavior Inventory Mania and Depression scales [22]) and other symptoms.

Participants were paid $5 for each monthly call with the study clinician and $5 for each monthly REDCap survey they completed. Additionally, they earned $0.50 per day that they complied with Beiwe (i.e., the data sync properly) and $0.50 for each of the thrice-weekly surveys they completed for up to $15.50 per month.

### Statistical analysis

A Shapiro-Wilk test was performed on all data to assess for normality. Descriptive statistics were calculated for data completeness and quality for the study. Demographic differences between participants who dropped out early and those who completed the full 18-month study were examined. Group differences (BD or TD) were tested using the Fisher exact test or Pearson’s Chi-square (parametric data), and Welch’s two-sample t-test or Mann Whitney U Test (nonparametric data). Statistical significance was set at p < 0.05. All analyses were conducted using R version 4.2.2.

## Results

### Sample characteristics

The average age at baseline was 15.85 years old (SD = 1.37). Approximately half (54%) of the sample identified as female, 46% identified as non-Hispanic White, 23% as Hispanic, 15% as non-Hispanic Asian/South Asian, 6.2% as non-Hispanic Black or African American, and 10% as Other. Nearly all participants (94%) used an iPhone. Mother (BD = 14.35 years, TD = 16.27 years, p = 0.018) and father education (BD = 13.64 years, TD = 15.82 years, p = 0.018) were higher in the typically developing group than the bipolar group. See Table 1.

**Table 1:** Sample characteristics.

Characteristic	Overall, N = 48^1^	Bipolar, N = 26	Typically Developing, N = 22	p-value^2^
Age, Mean (SD)	15.85 (1.37)	16.04 (1.34)	15.64 (1.40)	0.2287
Gender, n (%)				0.155
Female	26 (54%)	16 (62%)	10 (45%)	
Male	20 (42%)	8 (31%)	12 (55%)	
Other	2 (4.2%)	2 (7.7%)	0 (0%)	
Race and Ethnicity, n (%)				0.640
Hispanic	11 (23%)	6 (23%)	5 (23%)	
Non-Hispanic Black or African American	3 (6.2%)	3 (12%)	0 (0%)	
Non-Hispanic Asian/South Asian	7 (15%)	4 (15%)	3 (14%)	
Non-Hispanic White	22 (46%)	11 (42%)	11 (50%)	
Other	5 (10%)	2 (7.7%)	3 (14%)	
Dropout, n (%)	9 (19%)	5 (19%)	4 (18%)	>0.999
Duration of Participation in months, Mean (SD)	17.1 (3.7)	17.7 (2.9)	16.5 (4.5)	0.085
Phone Type, n (%)				>0.999
Android	2 (4.2%)	1 (3.8%)	1 (4.5%)	
iPhone	46 (96%)	25 (96%)	21 (95%)	
Father’s education, Mean (SD)	14.66 (3.19)	13.64 (2.94)	15.82 (3.13)	0.018
Mother’s education, Mean (SD)	15.23 (2.74)	14.35 (3.19)	16.27 (1.61)	0.018

1 Mean (SD); n (%).

2 Welch’s Two Sample t-test or Mann Whitney U Test; Fisher’s exact test.

### Retention

Thirty-nine of the 48 (81%) participants completed the full 18-month study. The average duration of participation was 17.1 (SD = 3.7) months. Among those who dropped out, five were in the bipolar group, and four were in the typically developing group. Average duration of participation was 17.2 (SD = 2.9) months for individuals in the bipolar group and 16.5 (SD = 4.5) months for those in the typically developing group. There were no differences between participants who completed the full 18-month study and those who dropped out. For further information, please see S1 Table.

### Data completeness

**Fig 1:**
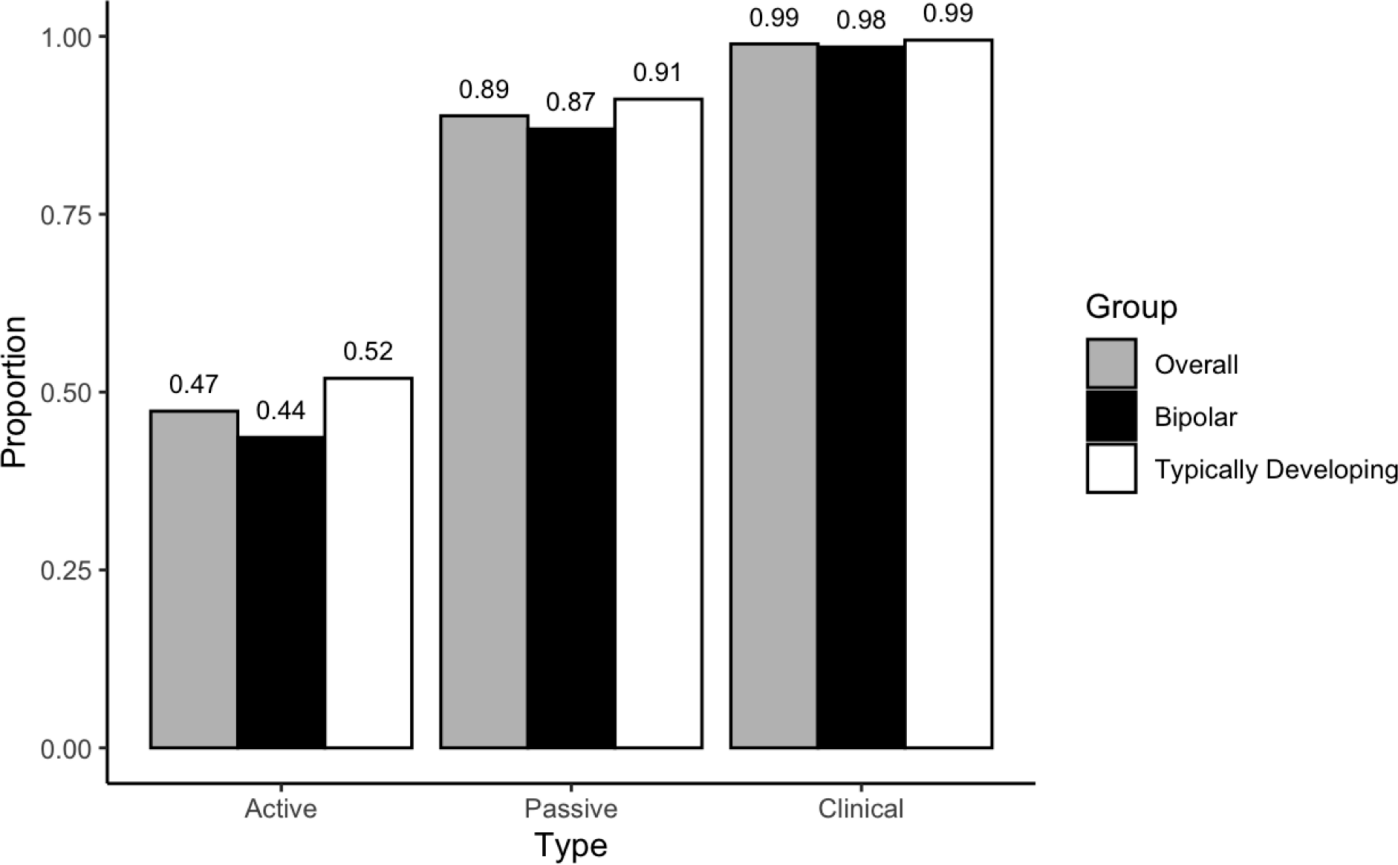
Completion rate by data collection method and group. Note. Data collection methods include Beiwe active survey data assessment (Active), Beiwe passive data collection (Passive), and clinical interview assessment (Clinical). Exact proportions are shown above bar.

Completion rates are shown in Fig 1. For the thrice-weekly surveys, average completion was 47% (4,945 out of 10,448 surveys delivered while participants were enrolled). Average passive data completion rate was 89% (22,233 out of 25,029 survey days). The average clinical interview completion rate was 99% (826 out of 835 interviews).

By group, the BD group had a slightly lower survey completion rate than the typically developing group (BD = 44% versus TD = 52%, χ^2^ = 71.454, p < 0.0001). Passive data completion rates (days data were collected) were lower for the BD group (BD = 87% versus TD = 91%, χ^2^ = 109.09, p < 0.0001). Clinical interview completion was similar for both (BD = 98% versus TD = 99%, χ^2^ = 1.8362, p = 0.1754).

**Fig 2:**
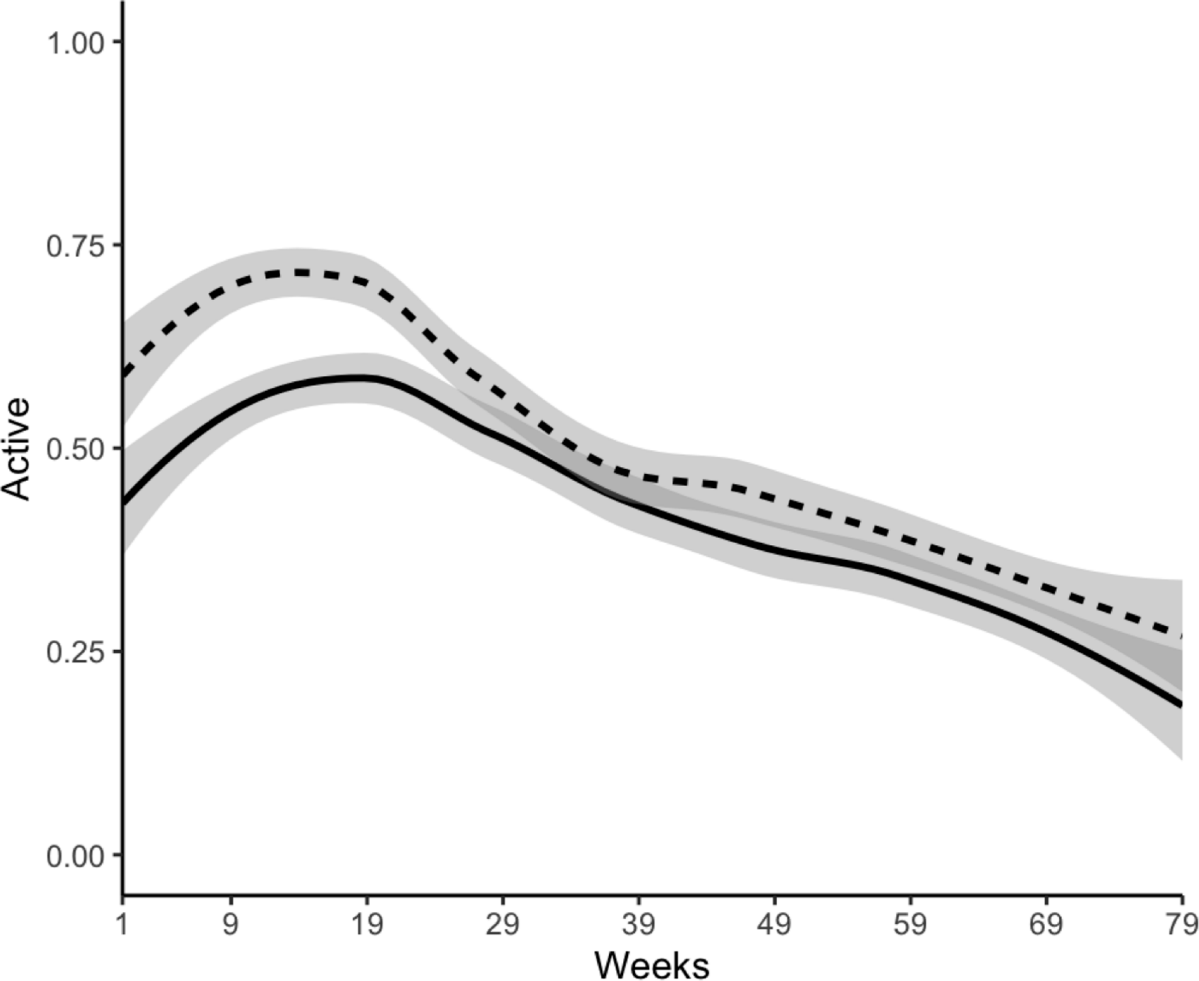
Data collection completion over time by group. Note. Participation procedure types include Beiwe active survey data assessment (Active), clinical interview assessment (Clinical), Beiwe passive data collection (Passive). Exact proportions are shown above bar. Completion rate for EMA and Clinical is proportion of number of surveys completed per month. Completion rate for Passive is proportion of days (>0 bytes) completed per month.

Although the proportion of days passive data were collected was consistent over time, both the clinical interviews and active survey completion decreased over the course of the study. For the active surveys, completion was 65% in the first 20 weeks, 52% in the second 20 weeks, 40% in the third 20 weeks, and 30% in the final 19 weeks. For the clinical interview data, completion was 98% in the first 4 months, 94% in the second 4 months, 92% in the third 4 months, 85% in the fourth 4 months, 82% in the last 3 months. The decline was more gradual for the clinical interviews compared to the active survey completions. For the passive data, completion was 90% in the first 20 weeks, 95% in the second 20 weeks, 95% in the third 20 weeks, and 94% in the final 19 weeks. See Fig 2.

Patterns of clinical interview completion were not different by the group. Group differences were observed for Beiwe survey completion over time. Although survey completion rates were higher for TD participants (TD = 70% vs. BD = 56%) in the earlier part of the study (<29 weeks), the rates become similar (TD = 41% vs. BD = 36%) over time. A similar pattern was observed for the passive data completion (earlier in study: TD = 93% and BD = 90% vs. later in study: TD = 95% vs. BD = 94%).

### Data quality

**Fig 3:**
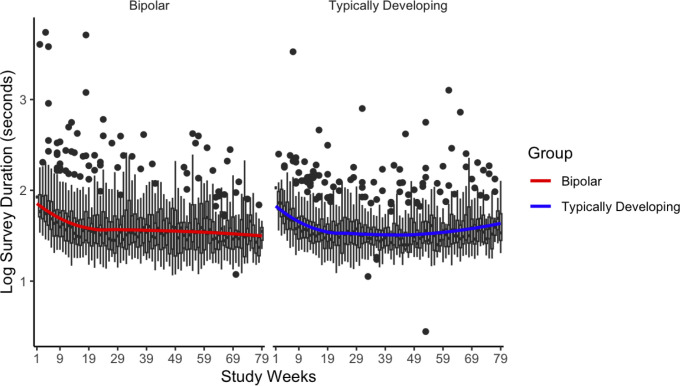
Survey duration over study period. Note. Survey duration was transformed to visualize the full-scale survey duration.

For the Beiwe surveys, average survey duration was 50.51 seconds. The median duration was 34.70 seconds and ranged from 2.78 seconds to 1.5 hours. Approximately 98% of responses fell within the acceptable response time (between >15 seconds and <5 minutes). There were more out of range response times among BD than TD (BD = 2.3% vs. TD = 0.9%, p < 0.001). We also examined the distribution of survey duration by study weeks (see Fig 3). For both groups, there was a decline in survey duration until week 20 of the study. After this, the survey duration appears to remain relatively stable, likely due to familiarity with the survey content. See S2 Table.

For selected passive data streams, the summary statistics are shown in S3 Table. For accelerometer data, the average data volume per person was 4,101 MB (SD = 2,091 MB, median = 3,865 MB, max = 8,822 MB). For GPS data, the average per person data volume was 120 MB (SD = 65 MB, median = 115 MB, max = 261 MB). For gyroscope data, the average per person data volume was 4,867 MB (SD = 2,182 MB, median = 4,991 MB, max = 9,704 MB). Statistical differences in the data volume were not observed between BD and TD groups. Data volume was highly variable across individuals; there was no discernable group difference in data volume collection over time. As a sensitivity analysis, we compared average data volume during the first year of the COVID-19 pandemic (March 2020-March 2021) to the following year. There were no consistent differences across data types.

## Discussion

The findings from this study support the potential of longitudinal mood monitoring using smartphone-based digital phenotyping in adolescents. This is the first study to collect and report digital phenotyping data from adolescent patients and community controls followed for longer than a year; previous investigations of young people have been comparatively short [23,24]. The second major finding is that the quality of passively collected phone sensor data was high and more complete than the other forms of data (self-report surveys) typically collected between appointments in psychiatric settings. Although there were individual differences in data volume, the data collected were more than adequate to provide high resolution behavioral information. These findings suggest that if an association between passive data and clinically significant phenomena exist in adolescents, as has been found in adults [12,13,25,26], passive data could be a practical, nonburdensome way to monitor mental health in this population long-term.

Consistent with our initial hypothesis, participants exhibited high adherence to the digital phenotyping protocol throughout the 18-month study period. Completion rates across data types were notably high, with clinical interviews achieving an impressive 99%, passive data reaching 89%, and thrice-weekly surveys at 47%. The Beiwe survey completion is consistent with the findings of previous studies [27–29]. Of note, participants received reminders of their clinical interviews and, as much as possible, these were rescheduled when missed. In contrast, participants received a notification through the Beiwe app to complete their surveys, but were not reminded directly by the study team. At the end of the month, participants received feedback with their payment, letting them know how many surveys they had missed. Metadata (i.e., volume) for the passive data streams were monitored by the study team and participants were reminded to log into the app when no data appeared over multiple days. Importantly, this is a process that could be automated to further reduce the resources necessary to collect these data. Although surveys have many appealing qualities, including relative ease of interpretation, our study and others have demonstrated that they are not a viable option for frequent assessment over months or years due to declining adherence over time. For individuals with chronic mental health conditions, monitoring through digital phenotyping supports a flexible treatment approach that responds to changes in an individual’s mental health status. Tailored treatment offers people with BD the best chance at maintaining stable mood. Overall, our results indicate that digital phenotyping is a viable approach to long-term monitoring and that it requires less time and resources than traditional approaches.

Both the surveys and passive data had relatively high data quality. Survey duration serves as a valuable metric for us to gauge the thoughtfulness of a response. Individuals who answer each question within a second are unlikely to offer meaningful information. It took BD participants slightly longer to complete the surveys, on average (BD 56 seconds vs. TD 45 seconds). This is consistent with other studies [30] and may be indicative of differences in cognitive function between the two groups [31]. Interestingly, the response time fell initially and then stabilized, suggesting that participants were able to answer more quickly as they became very familiar with the questions asked. There were no significant group differences in the quality of passive data, though BD participants had data from fewer days and there was variability in data quality across individual. This could be due to phone operating system differences, space availability on the individual’s phone, or other factors.

Although the results of this study suggest that digital phenotyping has significant potential as a method of long-term mental health monitoring, there are aspects of our approach that are unique to research that may have implications for the use of digital phenotyping clinically. Perhaps most significant is that our participants were paid for their participation. Although the amounts they earned for the passive data was low (up to $1.50 per week), in a clinical setting, no compensation would be offered. It is possible that this would have a detrimental effect on patient adherence with passive data collection. However, if these data were collected in a clinical context, it would be done to support the patient’s treatment goals. We expect that hearing about the benefits of the data from one’s provider and observing how one’s passive data relate to mental health outcomes would motivate patients even more than the small sums of money they received in our study. We also had regular phone calls with the participants and got to know them over the 18-month course of the study, which may have increased their motivation to maintain the app. In a clinical setting, patients would also have relationships with clinicians and staff, but the level of interaction necessary to ensure accountability is not clear. Related, when there are app modifications or phone operating system upgrades, patients may need to reregister their app or take other steps for the app to continue working properly, as would be the case for most other smartphone applications. For long-term monitoring to be effective, a member of the clinic staff would need to monitor data and work with patients to maintain app functionality.

It is important to consider the ethical implications of digital phenotyping. Digital phenotyping involves the collection of sensitive data, including location, making data security and privacy of utmost importance. There have been increasing efforts by the government [32] and field experts to develop guidelines and regulatory framework for data collection and analytics, but these are not yet standardized [33,34]. Additionally, participants’ and patients’ behavior may be influenced by the awareness that they are being observed or monitored, also known as the “Hawthorne effect” [35,36]. However, this effect is arguably small, as even in studies where the observed behavior is related to performance the observed effects tend to be small and deteriorate rapidly (e.g., in a study observing healthcare workers’ compliance with cleanliness protocols the effect was gone less than an hour after overt observance ended [37,38]). Finally, when clinics or research institutions have data that may indicate clinical risk for patients or participants, it is important to consider their responsibility for acting on that information.

This study has several limitations. First, data collection is ongoing, and the current preliminary findings represent only 48 participants. Second, this is a unique cohort; most participants completed part of the data collection during the initial COVID-19 pandemic period (i.e., lockdown), which may have impacted the study engagement. It is difficult to know what the nature of this impact was; participants may have spent more time on their phones in the absence of other activities during lockdown, but many families experienced high stress, and fewer youth were brought for inpatient or outpatient psychiatric care at our facility, both of which could negatively impact enrollment and engagement. Importantly, there were no differences in data completeness in sensitivity analyses comparing participants enrolled in 2020 or after. Related, not every person approached about participating in the study consented; those who agreed may be more interested/engaged with digital technology.

## Conclusion

The present study contributes valuable insights into the feasibility, acceptability, and quality of digitally collected data for longitudinal monitoring in adolescents. The observed completion patterns and data quality support the potential integration of digital phenotyping as a valuable clinical and research tool for mental health monitoring, offering new possibilities for advancing our understanding of behavioral experiences. A necessary next step is to determine how well the digital data capture changes in mental health to determine the clinical utility of this approach. If digital data correspond well to traditional clinical data (e.g., interviews, self-report), digital phenotyping may be used to predict clinically-significant changes in mental health status and to support the use of personalized interventions and just-in-time treatment approaches to reduce the consequences of mental health disorders in adolescents.

## Supporting information

S1 Table Sample characteristics comparing participants who completed versus dropped out of the study. (DOCX)

S2 Table Summary of survey duration statistics. (DOCX)

S3 Table Passive data volume in megabytes by group. (DOCX)

## References

[1] Goldstein BI, Birmaher B, Carlson GA, DelBello MP, Findling RL, Fristad M. The International Society for Bipolar Disorders Task Force report on pediatric bipolar disorder: knowledge to date and directions for future research. Bipolar Disorders. 2017. 10.1111/bdi.12556 PMC5716873 28944987

[2] Elanjithara TE, Frangou S, McGuire P. Treatment of the early stages of bipolar disorder. Adv Psychiatric Treatment. 2011;17(4):283–91.

[3] Vieta E, Langosch JM, Figueira ML, Souery D, Blasco-Colmenares E, Medina E, et al. Clinical management and burden of bipolar disorder: results from a multinational longitudinal study (WAVE-bd). Int J Neuropsychopharmacol. 2013;16(8):1719–32. doi: 10.1017/S1461145713000278 23663490

[4] Yatham LN, Kennedy SH, Parikh SV, Schaffer A, Bond DJ, Frey BN, et al. Canadian Network for Mood and Anxiety Treatments (CANMAT) and International Society for Bipolar Disorders (ISBD) 2018 guidelines for the management of patients with bipolar disorder. Bipolar Disord. 2018;20(2):97–170. doi: 10.1111/bdi.12609 29536616 PMC5947163

[5] Merikangas KR, He J, Burstein M, Swendsen J, Avenevoli S, Case B, et al. Service utilization for lifetime mental disorders in U.S. adolescents: results of the National Comorbidity Survey-Adolescent Supplement (NCS-A). J Am Acad Child Adolesc Psychiatry. 2011;50(1):32–45. doi: 10.1016/j.jaac.2010.10.006 21156268 PMC4408275

[6] Owens PL, Hoagwood K, Horwitz SM, Leaf PJ, Poduska JM, Kellam SG, et al. Barriers to children’s mental health services. J Am Acad Child Adolesc Psychiatry. 2002;41(6):731–8. doi: 10.1097/00004583-200206000-00013 12049448

[7] Costello EJ, He J, Sampson NA, Kessler RC, Merikangas KR. Services for adolescents with psychiatric disorders: 12-month data from the National Comorbidity Survey-Adolescent. Psychiatr Serv. 2014;65(3):359–66. doi: 10.1176/appi.ps.201100518 24233052 PMC4123755

[8] Torous J, Kiang MV, Lorme J, Onnela J-P. New tools for new research in psychiatry: a scalable and customizable platform to empower data driven smartphone research. JMIR Ment Health. 2016;3(2):e16. doi: 10.2196/mental.5165 27150677 PMC4873624

[9] Onnela JP, Rauch SL. Harnessing smartphone-based digital phenotyping to enhance behavioral and mental health. Neuropsychopharmacology. 2016;41(7):1691–6. 26818126 10.1038/npp.2016.7 PMC4869063

[10] Onnela J-P. Opportunities and challenges in the collection and analysis of digital phenotyping data. Neuropsychopharmacology. 2021;46(1):45–54. doi: 10.1038/s41386-020-0771-3 32679583 PMC7688649

[11] Bufano P, Laurino M, Said S, Tognetti A, Menicucci D. Digital phenotyping for monitoring mental disorders: systematic review. J Med Internet Res. 2023;25:e46778. doi: 10.2196/46778 38090800 PMC10753422

[12] Barnett I, Torous J, Staples P, Sandoval L, Keshavan M, Onnela J-P. Relapse prediction in schizophrenia through digital phenotyping: a pilot study. Neuropsychopharmacology. 2018;43(8):1660–6. doi: 10.1038/s41386-018-0030-z 29511333 PMC6006347

[13] Ben-Zeev D, Brian R, Wang R, Wang W, Campbell AT, Aung MSH, et al. CrossCheck: integrating self-report, behavioral sensing, and smartphone use to identify digital indicators of psychotic relapse. Psychiatr Rehabil J. 2017;40(3):266–75. doi: 10.1037/prj0000243 28368138 PMC5593755

[14] Vaghela M, Sasidhar K, Parikh A, Wagani R. Assessing mobile usage, physical activity and sleep through smartphone sensing: a digital phenotype study. SN Comput Sci. 2022;3(5). doi: 10.1007/s42979-022-01221-x

[15] Harrer M, Adam SH, Baumeister H, Cuijpers P, Karyotaki E, Auerbach RP, et al. Internet interventions for mental health in university students: a systematic review and meta-analysis. Int J Methods Psychiatr Res. 2019;28(2):e1759. doi: 10.1002/mpr.1759 30585363 PMC6877279

[16] Seko Y, Kidd S, Wiljer D, McKenzie K. Youth mental health interventions via mobile phones: a scoping review. Cyberpsychol Behav Soc Netw. 2014;17(9):591–602. doi: 10.1089/cyber.2014.0078 25007383

[17] Birmaher B, Axelson D, Goldstein B, Strober M, Gill MK, Hunt J, et al. Four-year longitudinal course of children and adolescents with bipolar spectrum disorders: The course and outcome of bipolar youth (COBY) study. Am J Psychiatry. 2009;166(7):795–804. 19448190 10.1176/appi.ajp.2009.08101569 PMC2828047

[18] Onnela JP, Dixon C, Griffin K, Jaenicke T, Minowada L, Esterkin S. Beiwe: a data collection platform for high-throughput digital phenotyping. J Open Source Software. 2021;6(68):3417.

[19] Kiang MV, Chen JT, Krieger N, Buckee CO, Alexander MJ, Baker JT, et al. Sociodemographic characteristics of missing data in digital phenotyping. Sci Rep. 2021;11(1):15408. doi: 10.1038/s41598-021-94516-7 34326370 PMC8322366

[20] Axelson D, Birmaher BJ, Brent D, Wassick S, Hoover C, Bridge J, et al. A preliminary study of the Kiddie Schedule for Affective Disorders and Schizophrenia for school-age children mania rating scale for children and adolescents. J Child Adolesc Psychopharmacol. 2003;13(4):463–70. doi: 10.1089/104454603322724850 14977459

[21] Keller MB, Lavori PW, Friedman B, Nielsen E, Endicott J, McDonald-Scott P, et al. The longitudinal interval follow-up evaluation. A comprehensive method for assessing outcome in prospective longitudinal studies. Arch Gen Psychiatry. 1987;44(6):540–8. doi: 10.1001/archpsyc.1987.01800180050009 3579500

[22] Youngstrom EA, Frazier TW, Demeter C, Calabrese JR, Findling RL. Developing a 10-item mania scale from the Parent General Behavior Inventory for children and adolescents. J Clin Psychiatry. 2008;69(5):831–9. doi: 10.4088/jcp.v69n0517 18452343 PMC2777983

[23] Ren B, Balkind EG, Pastro B, Israel ES, Pizzagalli DA, Rahimi-Eichi H. Predicting states of elevated negative affect in adolescents from smartphone sensors: a novel personalized machine learning approach. Psychol Med. 2022:1–9. 10.1017/S0033291722002161 PMC10650966 35894246

[24] Jacobson NC, Summers B, Wilhelm S. Digital biomarkers of social anxiety severity: digital phenotyping using passive smartphone sensors. J Med Internet Res. 2020;22(5):e16875. doi: 10.2196/16875 32348284 PMC7293055

[25] Zulueta J, Piscitello A, Rasic M, Easter R, Babu P, Langenecker SA, et al. Predicting mood disturbance severity with mobile phone Keystroke Metadata: a biaffect digital phenotyping study. J Med Internet Res. 2018;20(7):e241. doi: 10.2196/jmir.9775 30030209 PMC6076371

[26] Faurholt-Jepsen M, Vinberg M, Frost M, Christensen EM, Bardram JE, Kessing LV. Smartphone data as an electronic biomarker of illness activity in bipolar disorder. Bipolar Disord. 2015;17(7):715–28. doi: 10.1111/bdi.12332 26395972

[27] Clegg-Kraynok M, Barnovsky L, Zhou ES. Real, misreported, and backfilled adherence with paper sleep diaries. Sleep Med. 2023;107:31–5. 37116434 10.1016/j.sleep.2023.04.011

[28] Glenn CR, Kleiman EM, Kearns JC, Santee AC, Esposito EC, Conwell Y. Feasibility and acceptability of ecological momentary assessment with high-risk suicidal adolescents following acute psychiatric care. J Clin Child Adolescent Psychol. 2020;51(1):1–17. 10.1080/15374416.2020.1741377 32239986

[29] Wen CKF, Schneider S, Stone AA, Spruijt-Metz D. Compliance with mobile ecological momentary assessment protocols in children and adolescents: a systematic review and meta-analysis. J Med Internet Res. 2017;19(4):e132. doi: 10.2196/jmir.6641 28446418 PMC5425774

[30] Raugh IM, James SH, Gonzalez CM, Chapman HC, Cohen AS, Kirkpatrick B, et al. Digital phenotyping adherence, feasibility, and tolerability in outpatients with schizophrenia. J Psychiatr Res. 2021;138:436–43. doi: 10.1016/j.jpsychires.2021.04.022 33964681 PMC8192468

[31] Joseph MF, Frazier TW, Youngstrom EA, Soares JC. A quantitative and qualitative review of neurocognitive performance in pediatric bipolar disorder. J Child Adolesc Psychopharmacol. 2008;18(6):595–605. doi: 10.1089/cap.2008.064 19108664 PMC2768898

[32] Food and Drug Administration. Digital health technologies for remote data acquisition in clinical investigations. Food and Drug Administration; 2021. https://www.fda.gov/regulatory-information/search-fda-guidance-documents/digital-health-technologies-remote-data-acquisition-clinical-investigations

[33] Martinez-Martin N, Greely HT, Cho MK. Ethical development of digital phenotyping tools for mental health applications: delphi study. JMIR Mhealth Uhealth. 2021;9(7):e27343. doi: 10.2196/27343 34319252 PMC8367187

[34] Nock MK, Kleiman EM, Abraham M, Bentley KH, Brent DA, Buonopane RJ, et al. Consensus statement on ethical & safety practices for conducting digital monitoring studies with people at risk of suicide and related behaviors. Psychiatr Res Clin Pract. 2021;3(2):57–66. doi: 10.1176/appi.prcp.20200029 34414359 PMC8372411

[35] Landsberger HA. Hawthorne revisited: a plea for an open city. Ithaca, N.Y.: Cornell University; 1957.

[36] Pizzoli SFM, Monzani D, Conti L, Ferraris G, Grasso R, Pravettoni G. Issues and opportunities of digital phenotyping: ecological momentary assessment and behavioral sensing in protecting the young from suicide. Front Psychol. 2023;14:1103703. doi: 10.3389/fpsyg.2023.1103703 37441331 PMC10333535

[37] McCambridge J, Witton J, Elbourne DR. Systematic review of the Hawthorne effect: new concepts are needed to study research participation effects. J Clin Epidemiol. 2014;67(3):267–77. doi: 10.1016/j.jclinepi.2013.08.015 24275499 PMC3969247

[38] Vaisman A, Bannerman G, Matelski J, Tinckam K, Hota SS. Out of sight, out of mind: a prospective observational study to estimate the duration of the Hawthorne effect on hand hygiene events. BMJ Qual Saf. 2020;29(11):932–8. doi: 10.1136/bmjqs-2019-010310 32152090

