# Supplementary material for: Design and feasibility of smartphone-based digital phenotyping for long-term mental health monitoring in adolescents
Source: PLOS Digit Health. 2025 Jul 1;4(7):e0000883. doi: 10.1371/journal.pdig.0000883 (PMC12212497; doi:10.1371/journal.pdig.0000883)
Supplement: S1 Table — (DOCX) [file pdig.0000883.s001.docx]

S1 Table. Sample characteristics comparing participants who completed versus dropped out of the study

| **Characteristic** | **Overall**, N = 48*^1^* | **Completed the Study**, N = 39 | **Dropped Out from Study**, N = 9 | **p-value***^2^* |
| --- | --- | --- | --- | --- |
| Group, n (%) |  |  |  | >0.9 |
| Bipolar | 26 (54%) | 21 (54%) | 5 (56%) |  |
| Typically Developing | 22 (46%) | 18 (46%) | 4 (44%) |  |
| Age, Mean (SD) | 15.85 (1.37) | 15.82 (1.35) | 16.00 (1.50) | 0.765 |
| Gender, n (%) |  |  |  | >0.9 |
| Female | 26 (54%) | 21 (54%) | 5 (56%) |  |
| Male | 20 (42%) | 16 (41%) | 4 (44%) |  |
| Other | 2 (4.2%) | 2 (5.1%) | 0 (0%) |  |
| Race and Ethnicity, n (%) |  |  |  | 0.2 |
| Hispanic | 11 (23%) | 7 (18%) | 4 (44%) |  |
| Non-Hispanic Black or African American | 3 (6.2%) | 3 (7.7%) | 0 (0%) |  |
| Non-Hispanic Asian/South Asian | 7 (15%) | 7 (18%) | 0 (0%) |  |
| Non-Hispanic White | 22 (46%) | 19 (49%) | 3 (33%) |  |
| Other | 5 (10%) | 3 (7.7%) | 2 (22%) |  |
| iPhone, n (%) |  |  |  | 0.3 |
| Android | 2 (4.2%) | 1 (2.6%) | 1 (11%) |  |
| iPhone | 46 (96%) | 38 (97%) | 8 (89%) |  |
| Government benefits (Y/N), n (%) | 5 (10%) | 4 (10%) | 1 (11%) | >0.9 |
| Father’s education, Mean (SD) | 14.66 (3.19) | 14.97 (3.35) | 13.33 (2.06) | 0.076 |
| Mother’s education, Mean (SD) | 15.23 (2.74) | 15.26 (2.83) | 15.11 (2.47) | 0.849 |
| *^1^* n (%); Mean (SD) | |  |  |  |
| *^2^* Fisher's exact test; Welch’s Two Sample t-test or Mann Whitney U Test | | | | |
