# Supplementary material for: Design and feasibility of smartphone-based digital phenotyping for long-term mental health monitoring in adolescents
Source: PLOS Digit Health. 2025 Jul 1;4(7):e0000883. doi: 10.1371/journal.pdig.0000883 (PMC12212497; doi:10.1371/journal.pdig.0000883)
Supplement: S2 Table — (DOCX) [file pdig.0000883.s002.docx]

S2 Table. Summary of survey duration statistics

| **Characteristic** | **Overall**, k = 11,216 | **Bipolar**, k = 6,286 | **Typically Developing**, k = 4,930 | **p-value***^1^* |
| --- | --- | --- | --- | --- |
| Survey Duration (seconds) | |  |  | 0.062 |
| Mean (SD) | 51 (148) | 56 (191) | 45 (81) |  |
| Median (IQR) | 35 (26, 50) | 36 (26, 53) | 34 (26, 48) | |
| Range | 3, 5,487 | 12, 5,487 | 3, 3,356 |  |
| Survey Quality | |  |  | <0.001 |
| Acceptable Response Duration | 4,882 (98%) | 2,475 (98%) | 2,407 (99%) |  |
| Unacceptable Response Duration^2^ | 81 (1.6%) | 59 (2.3%) | 22 (0.9%) |  |
| *^1^*  Mann Whitney U Test and Pearson's Chi-squared test  *^2^* Unacceptable Response Duration defined as very short (i.e., <15 seconds) or very long (i.e., >5 minutes) responses | | | | |
