# Supplementary material for: Design and feasibility of smartphone-based digital phenotyping for long-term mental health monitoring in adolescents
Source: PLOS Digit Health. 2025 Jul 1;4(7):e0000883. doi: 10.1371/journal.pdig.0000883 (PMC12212497; doi:10.1371/journal.pdig.0000883)
Supplement: S3 Table — (DOCX) [file pdig.0000883.s003.docx]

S3 Table. Passive data volume in megabytes by group

| **Characteristic** | **Overall**, N = 48 | **Bipolar**,  N = 26 | **Typically Developing**,  N = 22 | **p-value***^1^* |
| --- | --- | --- | --- | --- |
| Accelerometer | |  |  | 0.2 |
| Mean (SD) | 4,203 (2,116) | 3,786 (1,748) | 4,697 (2,432) | |
| Median (IQR) | 3,945 (3,074, 5,562) | 3,799 (2,858, 4,630) | 4,792 (3,305, 6,500) | |
| Range | 2, 8,822 | 2, 7,433 | 7, 8,822 |  |
| GPS |  |  |  | 0.087 |
| Mean (SD) | 123 (66) | 108 (54) | 142 (74) |  |
| Median (IQR) | 117 (77, 176) | 105 (74, 130) | 137 (94, 195) | |
| Range | 3, 261 | 3, 223 | 12, 261 |  |
| Gyro |  |  |  | 0.3 |
| Mean (SD) | 4,955 (2,187) | 4,669 (1,880) | 5,293 (2,506) | |
| Median (IQR) | 5,102 (3,911, 6,275) | 4,847 (4,252, 5,888) | 5,489 (3,890, 6,979) | |
| Range | 0, 9,704 | 0, 7,514 | 2, 9,704 |  |
| *^1^* Welch’s Two Sample t-test | |  |  |  |
